# Esophageal intramural metastasis from adenocarcinoma of esophagogastric junction: a case report and literature review

**DOI:** 10.3389/fonc.2026.1792292

**Published:** 2026-05-11

**Authors:** Yongfen Ma, Chen-Wei Pu, Yao Zheng, Xiaoyu Sun, Jingmei Cao

**Affiliations:** 1 Department of Gastroenterology, Zibo Central Hospital, Zibo, China; 2 Department of Pulmonary and Critical Care Medicine, Zibo Central Hospital, Zibo, China; 3 Department of Pathology, Zibo Central Hospital, Zibo, China

**Keywords:** adenocarcinoma of esophagogastric junction, cardia adenocarcinoma, case report, esophagectomy, gastrectomy, intramural metastasis

## Abstract

**Background:**

Intramural metastasis from adenocarcinoma of the esophagogastric junction (AEG) to the esophagus is a rare event and is associated with a poor prognosis.

**Case summary and literature review:**

We report a 77-year-old male patient diagnosed with AEG with esophageal intramural metastasis. Upper gastrointestinal endoscopy revealed an infiltrative ulcerative tumor at the esophagogastric junction and a submucosal protrusion in the middle esophagus. Biopsies from both lesions confirmed moderately to poorly differentiated adenocarcinoma. The patient received seven cycles of neoadjuvant chemotherapy (oxaliplatin and tegafur), followed by lower esophagectomy and total gastrectomy. Postoperative radiotherapy was administered for residual esophageal metastasis. Although the patient initially improved, he died approximately three years after surgery. A systematic search of PubMed, Embase, and Web of Science identified six relevant articles on esophageal intramural metastasis from AEG. The findings suggest that tumor cells primarily spread through venous and lymphatic channels within the esophageal wall. The presence of intramural metastasis on endoscopy indicates systemic disease and a poor prognosis. An individualized treatment approach, potentially combining neoadjuvant chemotherapy, radiotherapy, immunotherapy, and surgery, may offer the best outcome.

**Conclusion:**

Esophageal intramural metastasis from AEG is a rare but aggressive metastatic pattern. Its detection warrants prompt recognition and a multidisciplinary, individualized treatment strategy.

## 1 Introduction

Adenocarcinoma of the esophagogastric junction (AEG) is a malignancy arising from the epithelial lining of the esophagogastric junction (1). According to the NCCN Clinical Practice Guidelines in Oncology for Esophageal and Esophagogastric Junction Cancers, multidisciplinary team management is essential for all patients, with preoperative chemoradiation or perioperative chemotherapy followed by surgery representing standard curative-intent strategies for locally advanced resectable disease (2). In China, a nationwide cohort study involving 220,304 patients reported that AEG accounted for 38.16% of all gastric adenocarcinomas, with a 5-year overall survival rate of 33.53% (3). The metastatic pattern of AEG significantly influences prognosis, staging, and treatment decisions, with common metastatic sites including lymph nodes, liver, peritoneum, lungs, bones, and brain (4, 5). Esophageal intramural metastasis (IM) represents a distinct form of tumor spread characterized by skip lesions within the esophagus. However, intramural metastasis of AEG to the esophagus is rarely reported and is associated with a poor prognosis. Herein, we present a case of AEG with concurrent intramural esophageal metastasis and provide a review of the relevant literature.

## 2 Case presentation

**Figure 1:**
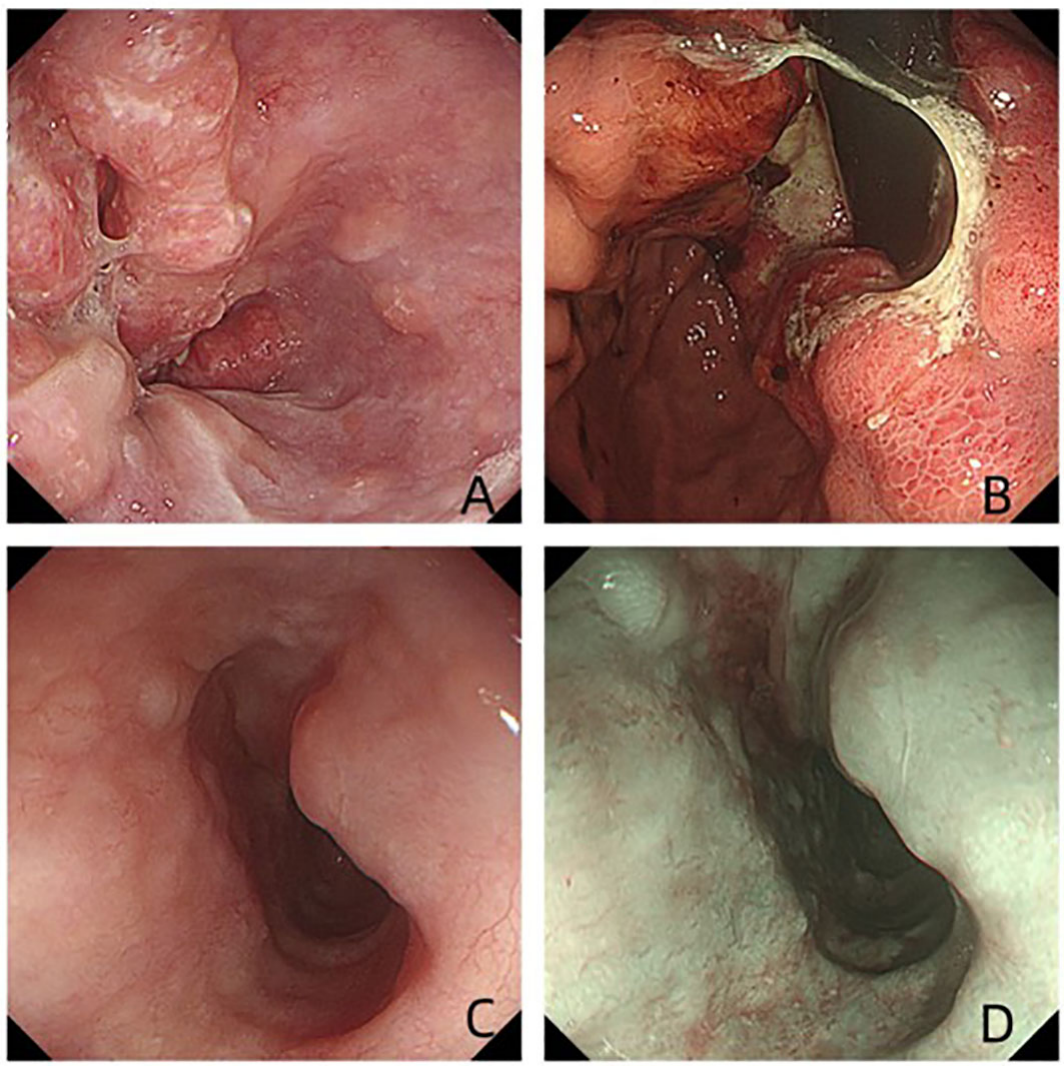
Endoscopic view of the ulcerated lesion **(A, B)**, Endoscopic view of the esophageal intramural metastasis **(C, D)**.

**Figure 2:**
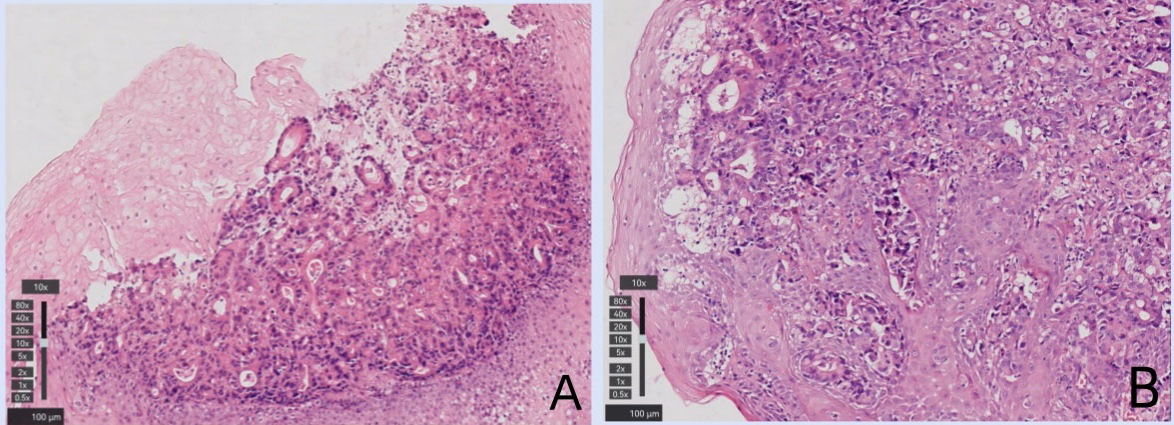
Histologic appearance of the esophageal intramural metastasis which located at 27 cm **(A)** from the incisors also revealed moderately and poorly differentiated adenocarcinoma under the mucosa. Histologic appearance of the primary tumor of the cardia, which demonstrated moderately and poorly differentiated adenocarcinoma **(B)**.

**Figure 3:**
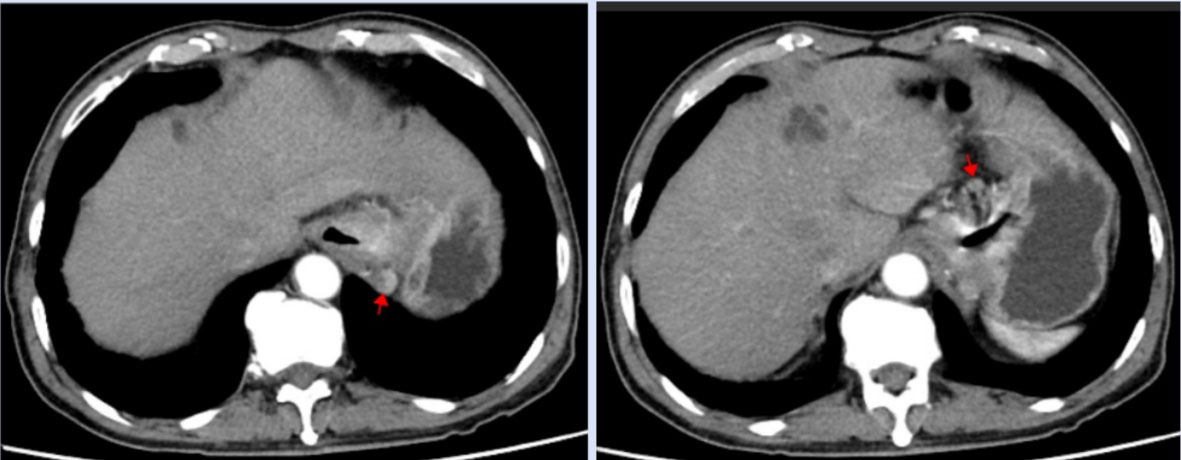
CT scan revealed multiple enlarged lymph nodes around the stomach.

A 77-year-old man with a two-month history of fatigue was admitted to the Department of Gastroenterology, ZiBo Central Hospital. He had a 50-year smoking history and an 8-year history of hypertension and coronary heart disease. Routine laboratory tests revealed mild anemia. Upper gastrointestinal endoscopy showed an infiltrative, ulcerating tumor at the esophagogastric junction. A mound-shaped protrusion (~0.7 cm in diameter) was found in the esophagus 27 cm from the incisors (**Figure 1**), with normal mucosa between the two lesions. Biopsies were taken from both sites. The ulcerated lesion was confirmed as moderately to poorly differentiated adenocarcinoma, and the esophageal submucosal mass showed identical histology beneath the squamous epithelium (**Figure 2**). Contrast-enhanced CT revealed multiple enlarged perigastric lymph nodes (**Figure 3**). No carcinoma cells were identified at the esophageal margin or the duodenal bulb margin. The patient was diagnosed with AEG (cT3N1M0, stage IIIB) with intramural esophageal metastasis (Siewert type II).

**Figure 4:**
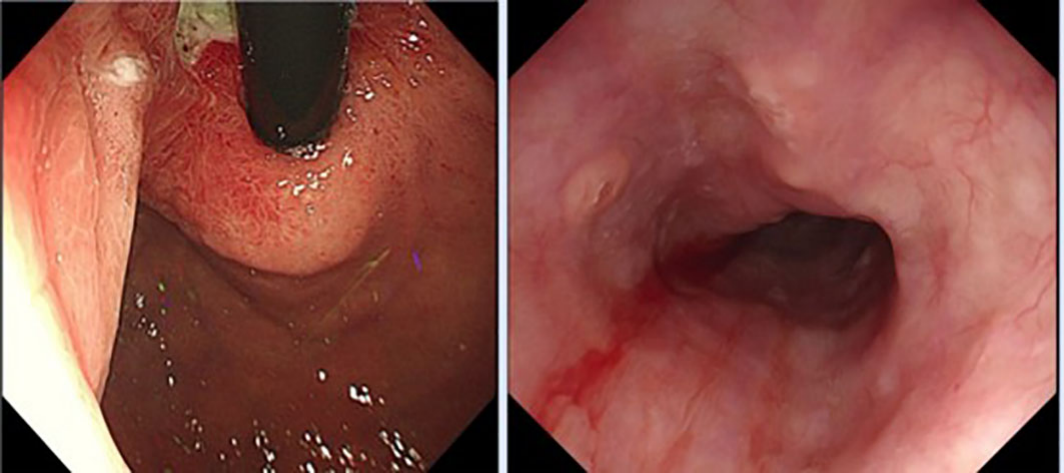
The follow-up gastroscopy revealed that both lesions had shrunk compared to before.

A preoperative multidisciplinary meeting was conducted involving the departments of Gastrointestinal Surgery, Oncology, and Thoracic Surgery. Given the locally advanced tumor stage (cT3N1M0) with intramural metastasis and regional lymph node involvement, neoadjuvant chemotherapy was recommended to downstage the tumor and reduce surgical trauma, thereby improving postoperative quality of life. The patient subsequently received seven cycles of the SOX regimen (oxaliplatin plus tegafur), with tegafur administered at a dose of 1 g/day for 5 days per cycle, and oxaliplatin administered at a dose of 200 mg per cycle on day 1 of each cycle. This regimen was selected based on the CSCO Clinical Guidelines for Gastric Cancer and supported by a phase III trial demonstrating its efficacy and favorable tolerability in advanced gastric/EGJ adenocarcinoma (6). After completion of chemotherapy, follow-up gastroscopy showed shrinkage of both lesions (**Figure 4**). No significant change in tumor stage was observed. Approximately six months after diagnosis, the patient underwent lower esophagectomy and total gastrectomy via right thoracotomy and laparotomy. Considering postoperative quality of life, only 2 cm of the distal esophagus was resected; the upper esophageal submucosal metastasis was not removed, as it had significantly shrunk after neoadjuvant chemotherapy and complete resection would have required an extended approach with greater trauma.

**Figure 5:**
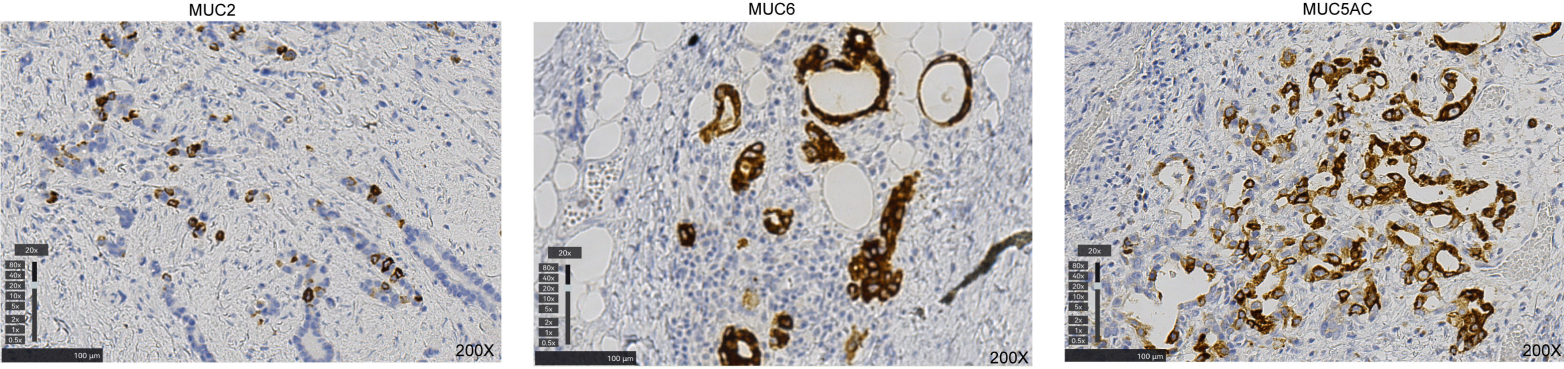
Immunohistochemical staining for mucin core proteins in the poorly differentiated adenocarcinoma. MUC2 shows focal positivity; MUC6 is positive in approximately 10% of tumor cells; and MUC5AC is positive in approximately 30% of tumor cells.

**Figure 6:**
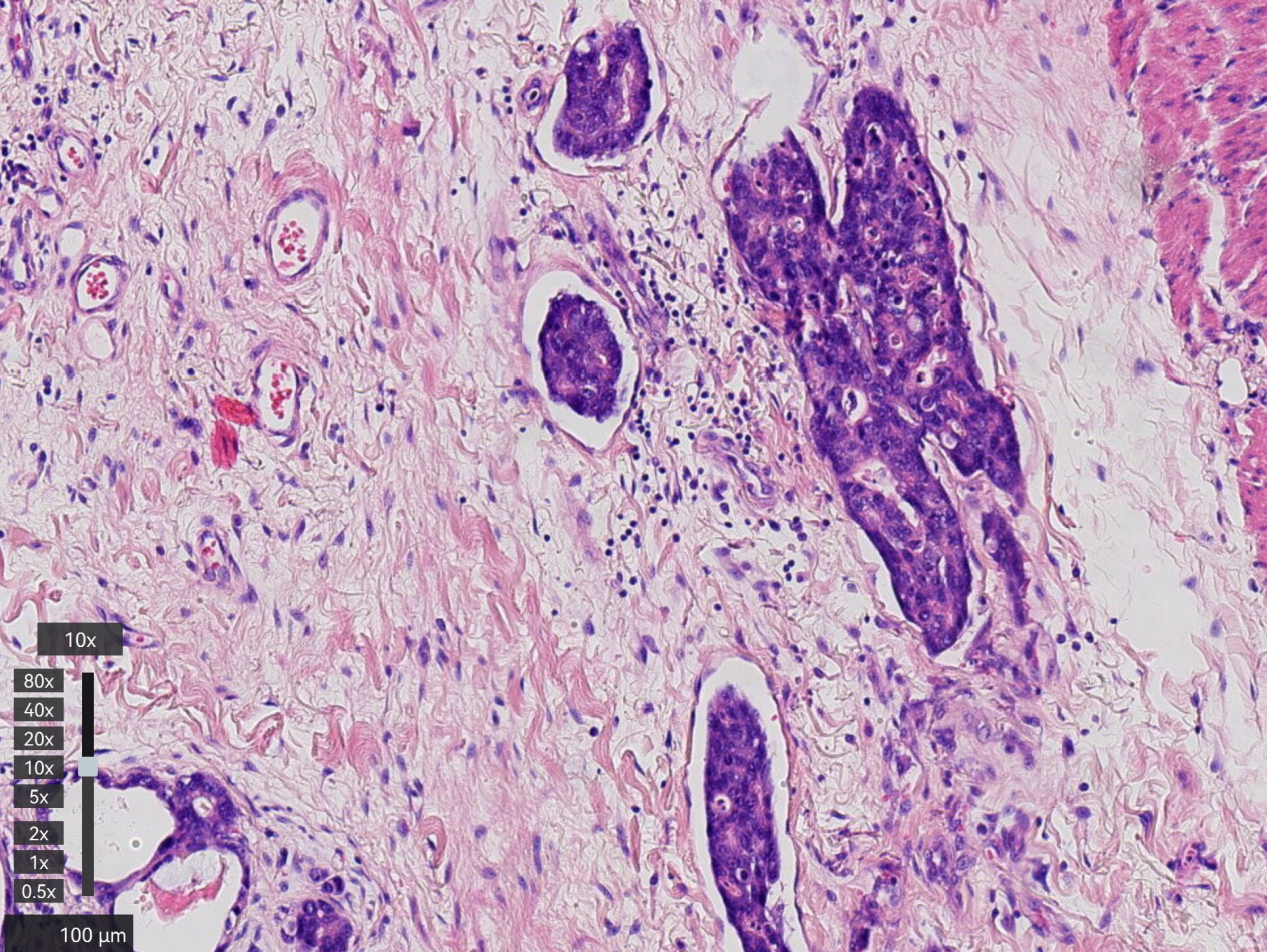
Tumor thrombus was identified within vascular channels in the resection specimen.

Histopathological examination confirmed a poorly differentiated adenocarcinoma. Immunohistochemistry revealed focal positivity for MUC2, MUC6 positivity in approximately 10% of tumor cells, and MUC5AC positivity in approximately 30% of tumor cells (**Figure 5**). The expression patterns of these mucin core proteins help characterize the tumor’s differentiation lineage, with MUC5AC and MUC6 indicating gastric phenotype and MUC2 indicating intestinal phenotype. Their co-expression in this case reflects the mixed histological features commonly observed in AEG. Moreover, All four mismatch repair (MMR) core proteins (MLH1, MSH2, MSH6, PMS2) were positive. PD-L1 expression was assessed by immunohistochemistry (clone 22C3), with a Combined Positive Score (CPS) of 2. HER-2 immunohistochemistry showed a negative result (score 1+). The tumor invaded through the serosa with marked lymph node metastasis. Multiple metastatic deposits were observed outside the serosa, and vascular tumor thrombus was identified (**Figure 6**). The postoperative pathological stage was ypT4aN3aM0, stage IIIC.

**Figure 7:**
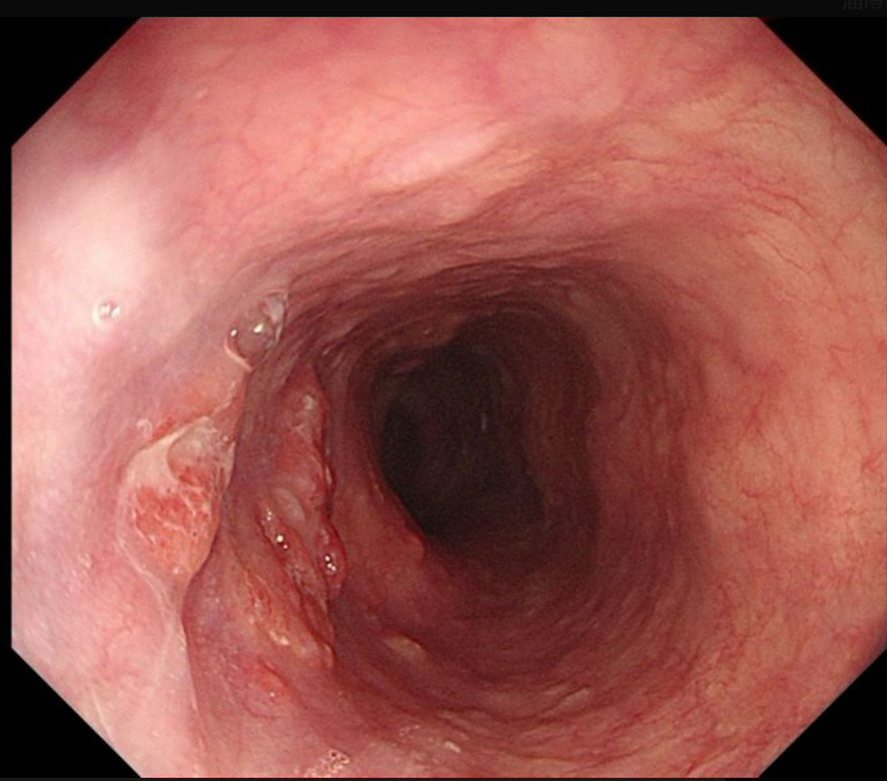
Re-examination of endoscopy revealed progression of the submucosal lesion in the esophagus.

Three months after surgery, follow-up gastroscopy revealed progression of the intramural esophageal metastasis (**Figure 7**). Adjuvant radiotherapy (with a total dose of 50 Gy) was selected instead of chemotherapy because the patient developed a postoperative anastomotic leak that precluded systemic chemotherapy. Immunotherapy and targeted therapy were considered but declined for financial reasons. Under gastroscopy, the lesion was marked with titanium clips to guide precise radiotherapy. The patient did not undergo further gastroscopy after radiotherapy, but his condition remained stable. Unfortunately, the disease progressed, and his overall health deteriorated. He died approximately three years after surgery.

## 3 Literature review

Esophageal intramural metastasis from esophagogastric junction (EGJ) adenocarcinoma is a rare metastatic route, reported only in sporadic case reports and small case series. To analyze the characteristics of these cases, we systematically searched PubMed, Embase, and Web of Science from inception to July 10, 2025. A total of 2,570 articles were initially identified. After applying predefined inclusion and exclusion criteria (**Supplementary Table 1**) and a structured study selection process (**Supplementary Figure 1**), six studies reporting esophageal intramural metastasis from EGJ adenocarcinoma were included (7–12). Quality assessment of the included studies was performed based on the CARE guidelines (**Supplementary Table 2**). Patient-level clinical characteristics, metastatic patterns, treatments, and outcomes are summarized in **Supplementary Table 3**, with descriptive statistics presented in **Supplementary Table 4**. Due to the small sample size and heterogeneity of case reports, no formal statistical analysis was performed.

To compare the characteristics of intramural metastasis (IM) between esophageal squamous cell carcinoma (ESCC) and AEG, we additionally identified three case reports (13–15)] comprising four ESCC patients with gastric IM. Quality assessment was performed using the CARE guidelines (**Supplementary Table 5**). Individual patient data on demographics, histology, metastatic sites, treatment modalities, and follow-up outcomes are detailed in **Supplementary Table 6**, while aggregated clinical characteristics are provided in **Supplementary Table 7**.

A comparative analysis of key clinical characteristics between the ESCC-IM group (n=4) and the EGJ adenocarcinoma-IM group (n=11) is presented in **Table 1**. Both groups showed a male predominance, although all EGJ patients were male (100%) compared with 75% in the ESCC group. Median age was similar (67.5 vs. 64 years). Solitary metastasis was observed in all ESCC patients (100%), whereas multiple metastases were more common in the EGJ group (55% vs. 0%). Regarding treatment, surgery alone was the mainstay for EGJ patients (73%), whereas half of the ESCC patients received chemoimmunotherapy with or without conversion surgery. Despite similarly high rates of lymph node metastasis in both groups (82% vs. 75%), the prognosis differed markedly between the two tumor types. In the EGJ group, overall mortality was 82% with a median overall survival of 8.5 months. In contrast, no deaths were reported in the ESCC group during a median follow-up of 7.5 months.

**Table 1:** Comparison of key clinical characteristics between patients with intramural metastasis from esophageal squamous cell carcinoma and those from adenocarcinoma of the esophagogastric junction (EGJ).

Characteristic	ESCC with IM group (n=4)	EGJ AC with IM group (n=11)
Demographics		
Male sex, n (%)	3 (75.0)	11 (100)
Age (years), median (range)	67.5 (59–76)	64 (52–74)
Intramural metastasis		
Solitary metastasis, n (%)	4 (100)	5 (45)
Multiple metastases, n (%)	0 (0)	6 (55)
Treatment		
Surgery alone, n (%)	2 (50.0)	8 (73)
Chemoimmunotherapy (± conversion surgery), n (%)	2 (50.0)	0 (0)
Prognosis		
Lymph node metastasis positive, n (%)	3 (75.0)	9 (82)
Overall mortality, n (%)	0 (0)	9 (82)
Median follow-up / overall survival (months), range	7.5 (7–8)*	8.5 (1.5–20)†

SCC, Esophageal squamous cell carcinoma; EGJ, Esophagogastric junction; AC, Adenocarcinoma; IM, Intramural metastasis.

*: Median follow-up time.

†: Median overall survival.

## 4 Discussion

AEG is a malignancy arising at the esophagogastric junction, with increasing global incidence (16, 17). Due to its unique location, AEG exhibits distinct biological behaviors, including higher invasiveness and risk of early metastasis compared to distal gastric cancer (18).

Esophageal intramural metastasis (IM) is a rare metastatic pattern in AEG (19). In our case, the metastatic focus presented as a submucosal tumor histologically identical to the primary lesion, with evidence of lymphatic invasion. The mechanism of IM involves both lymphatic and venous routes (20, 21). The rich lymphatic network within the esophageal wall provides the anatomic basis for intramural spread (22). Most reported AEG-IM cases show extensive lymphatic invasion and regional lymph node metastases (11), supporting lymphatic dissemination as the primary route. However, Hiramoto et al. (10) demonstrated that submucosal venous invasion may also occur. Thus, tumor cells can spread via both pathways.

The risk factors for AEG remain incompletely understood. In contrast to esophageal squamous cell carcinoma, which has well-established risk factors such as smoking and alcohol consumption, AEG is more closely associated with gastroesophageal reflux disease, Barrett’s esophagus, and obesity (23, 24) In our case, the positive immunostaining for MUC5AC and MUC6 (gastric phenotype) together with MUC2 (intestinal phenotype) suggests a mixed adenocarcinoma, reflecting the chronic inflammatory background often seen in AEG. Among the included cases, the majority were moderately or poorly differentiated, suggesting that AEG with intramural metastasis may exhibit more aggressive histological features.

A comparative analysis between AEG-associated IM and ESCC-associated IM reveals important differences that merit discussion. In our literature review, we identified four ESCC patients with gastric IM from three case reports. Both groups showed male predominance, but several distinctions emerged. In the AEG-IM group (n=11), multiple metastases were more common (55% vs. 0% in ESCC-IM), and overall mortality was substantially higher (82% vs. 0% during follow-up). These differences may reflect fundamental biological distinctions between the two tumor types. ESCC arises from squamous epithelium and is strongly associated with risk factors such as smoking and alcohol consumption, whereas AEG develops from glandular epithelium, often in the setting of Barrett’s esophagus and chronic gastroesophageal reflux (25). The higher mortality observed in AEG-IM patients may be attributable to the more aggressive nature of AEG, its propensity for early lymphatic invasion, and the advanced stage at diagnosis. Nevertheless, the small sample size (n=4 for ESCC-IM) precludes definitive conclusions, and larger comparative studies are needed.

Endoscopic ultrasound (EUS) is valuable for diagnosing IM but was underutilized in reported cases, including ours. EUS can determine the layer of origin (typically submucosal) (26), assess invasion depth with high accuracy (27), evaluate regional lymph nodes (28), and guide fine-needle aspiration for cytological confirmation (29). We recommend routine EUS evaluation when a submucosal lesion is encountered in AEG patients.

Distinguishing true intramural metastasis from implantation metastasis and multiple primary tumors is clinically important, as each entity carries different implications for staging and treatment. True intramural metastasis is characterized by tumor cells spreading via lymphatic or venous channels within the esophageal wall, typically presenting as a submucosal lesion with normal intervening mucosa; histological concordance with the primary tumor and evidence of vascular invasion further support this diagnosis (11, 12). In contrast, implantation metastasis occurs when tumor cells shed from the primary lesion implant onto the esophageal mucosa, a process that may be facilitated by gastroesophageal reflux or prior endoscopic manipulation; these lesions are usually polypoid and located on the mucosal surface rather than within the submucosa (30, 31). Multiple primary tumors represent synchronous independent neoplasms; while clonality analysis is the definitive method to distinguish them from IM, the absence of such testing makes identical histology and immunohistochemical profiles the key supportive features for IM (32, 33). In our case, the submucosal location of the esophageal lesion, its histological identity to the primary AEG, and the presence of lymphatic invasion collectively favor the diagnosis of true intramural metastasis over the other two entities.

Esophageal IM affects proximal margin determination. Barbour et al. (34) showed that a proximal margin >3.8 cm (ex vivo) is associated with improved survival in gastroesophageal junction adenocarcinoma. In our case, only 2 cm of distal esophagus was resected due to concerns about surgical trauma, which may have contributed to disease progression. Intramural metastasis can extend up to 7.7 cm orally from the primary tumor, with an average spread distance of 3.4 cm (35). Therefore, when IM is identified preoperatively, we suggest aiming for an R0 resection with an adequate proximal margin of normal esophagus, guided by intraoperative frozen section when necessary.

A key question is whether esophageal IM should be classified as M1 (distant metastasis) or locoregional disease (36). In our review, AEG-IM patients had high rates of lymph node metastasis (82%) and mortality (82%), suggesting that IM may be a marker of systemic dissemination. Hirota et al. (12) proposed that IM indicates systemic disease spread. While the current AJCC staging system does not explicitly classify IM as M1 (37), we suggest that IM should be considered a high-risk feature warranting intensified multimodal therapy.

Optimal treatment for AEG with IM remains undefined. Our patient received neoadjuvant chemotherapy (SOX regimen), surgery, and postoperative radiotherapy, achieving approximately three years of survival—favorable compared to the median of ~15 months reported in the literature. Recent studies support the effectiveness of neoadjuvant chemotherapy, radiotherapy, and immunotherapy for AEG (38). We recommend a multimodal, individualized approach.

In conclusion, intramural metastasis is a rare metastatic pattern of AEG. Tumor cells primarily disseminate to the esophageal wall via intramural veins and lymphatic vessels. The presence of esophageal intramural metastasis on endoscopy may suggest an increased risk of systemic spread and is often associated with a poor prognosis. However, given the limited number of reported cases, further studies are needed to better understand its clinical implications. Therefore, when such lesions are detected, a comprehensive multidisciplinary evaluation is warranted, and an individualized treatment approach—potentially combining neoadjuvant chemotherapy, radiotherapy, immunotherapy, and surgery—should be considered.
