# Supplementary material for: Esophageal intramural metastasis from adenocarcinoma of esophagogastric junction: a case report and literature review
Source: Front Oncol. 2026 May 11;16:1792292. doi: 10.3389/fonc.2026.1792292 (PMC13199032; doi:10.3389/fonc.2026.1792292)
Supplement: Supplementary file 1 [file Table1.docx]

| **Category** | **No.** | **Criterion** |
| --- | --- | --- |
| **Inclusion** | 1 | Published case reports or case series (including retrospective or prospective case series). |
|  | 2 | Patients with histologically or cytologically confirmed adenocarcinoma of the esophagogastric junction. |
|  | 3 | Definite evidence of esophageal intramural metastasis, clearly described or supported by imaging and/or pathological findings. |
|  | 4 | Complete case data available, including clinical characteristics, pathological diagnosis, metastatic sites, treatment details, and follow-up information, with extractable core data. |
| **Exclusion** | 1 | Non-case-report or non-case-series studies (e.g., basic research, reviews, systematic reviews, meta-analyses of overlapping case data, conference abstracts, editorials, short communications, etc.). |
|  | 2 | Diagnosed only as esophageal cancer or gastric cancer without clear pathological localization to the esophagogastric junction/cardia. |
|  | 3 | No evidence of esophageal intramural metastasis; only extraesophageal invasion, distant organ metastasis, or lymph node metastasis reported without mention of intramural metastasis. |
|  | 4 | Duplicate publications, overlapping data, or multiple reports of the same case; only the most recent or most complete version retained, others excluded. |
|  | 5 | Severe lack of data, precluding extraction of core clinical and pathological information. |
|  | 6 | Cases with another severe malignancy where the other tumor is the primary focus of the study. |

**Supplementary Table S1. Criteria for inclusion and exclusion of studies in this review**
