# Supplementary material for: Esophageal intramural metastasis from adenocarcinoma of esophagogastric junction: a case report and literature review
Source: Front Oncol. 2026 May 11;16:1792292. doi: 10.3389/fonc.2026.1792292 (PMC13199032; doi:10.3389/fonc.2026.1792292)
Supplement: Supplementary file 2 [file Table2.docx]

| **Author (Year)** | **Patient information** | **Clinical findings** | **Diagnostic assessment** | **Therapeutic interventions** | **Follow-up outcomes** | **Discussion quality** | **Informed consent** | **Patient perspective** | **Overall quality** |
| --- | --- | --- | --- | --- | --- | --- | --- | --- | --- |
| Hirota et al. (1998)^[7]^ | Comprehensive | Not described | Thorough | Well-documented | Reported | Relevant | Not mentioned | Not mentioned | High |
| Brotherton et al. (2022)^[8]^ | Comprehensive | Not described | Thorough | Well-documented | Reported | Relevant | Obtained | Not mentioned | High |
| Kurihara et al. (2006)^[9]^ | Comprehensive | Not described | Thorough | Well-documented | Reported | Relevant | Not mentioned | Not mentioned | High |
| Szántó et al. (2002)^[10]^ | Comprehensive | Not described | Thorough | Well-documented | Reported | Relevant | Not mentioned | Not mentioned | High |
| Hiramoto et al. (2017)^[11]^ | Comprehensive | Detailed | Thorough | Well-documented | Reported | Relevant | Not mentioned | Partially mentioned | High |
| Akamaru et al. (2018)^[12]^ | Comprehensive | Not described | Thorough | Well-documented | Reported | Relevant | Not mentioned | Not mentioned | High |

**Supplementary Table S2. Quality assessment of included case reports based on the** **CARE guidelines**
