# Supplementary material for: Esophageal intramural metastasis from adenocarcinoma of esophagogastric junction: a case report and literature review
Source: Front Oncol. 2026 May 11;16:1792292. doi: 10.3389/fonc.2026.1792292 (PMC13199032; doi:10.3389/fonc.2026.1792292)
Supplement: Supplementary file 3 [file Table3.docx]

**Supplementary Table S3. Clinical characteristics, metastatic patterns, treatment modalities, and outcomes of individual patients with esophageal intramural metastasis from adenocarcinoma of the esophagogastric junction (EGJ).**

| **Case** | **Reference** | **Age/Sex** | **Histology** | **No. of mets** | **Location** | **LN mets** | **Treatment** | **Survival (mo)** | **Outcome** |
| --- | --- | --- | --- | --- | --- | --- | --- | --- | --- |
| 1 | Hirota (1998)^[7]^ | 65/M | Moderate | 1 | 2 cm from EGJ | Yes | Surgery | 9 | Died |
| 2 | Brotherton (2022)^[8]^ | 74/M | Poor | Multiple | Mid-esophagus | No | CRT→chemo | — | Progression |
| 3 | Kurihara (2006)^[9]^ | 53/M | Poor | 3 | 0.5/4/7.2 cm | Yes | Surgery + S-1 | 20 | Died |
| 4 | Szántó (2002)^[10]^ | 67/M | ADC | 1 | 20 mm | Yes | Surgery | — | Alive |
| 5 |  | 52/M | ADC | 2 | 50 mm | Yes | Palliative | 3.3 | Died |
| 6 |  | 64/M | ADC | 2 | 20 mm | Yes | Surgery | 8.2 | Died |
| 7 |  | 67/M | ADC | 3 | 50 mm | Yes | Surgery | 0.7 | Died |
| 8 |  | 67/M | ADC | 1 | 50 mm | Yes | Palliative | 5.5 | Died |
| 9 |  | 57/M | ADC | 2 | 40 mm | Yes | Palliative | 3.9 | Died |
| 10 | Hiramoto (2017)^[11]^ | 66/M | ADC | 1 | 60 mm from EGJ | Yes | Surgery | 10+ | Alive |
| 11 | Akamaru (2018)^[12]^ | 58/M | Poor | 6 | 80 mm from EGJ | Yes | SP→ surgery | 15 | Died |

M, male; ADC, adenocarcinoma; CRT, chemoradiotherapy; LN, lymph node; mo, months; EGJ, esophagogastric junction; SP, S-1 + cisplatin.
