# Supplementary material for: Esophageal intramural metastasis from adenocarcinoma of esophagogastric junction: a case report and literature review
Source: Front Oncol. 2026 May 11;16:1792292. doi: 10.3389/fonc.2026.1792292 (PMC13199032; doi:10.3389/fonc.2026.1792292)
Supplement: Supplementary file 4 [file Table4.docx]

| **Characteristic** | **Summary statistic** |
| --- | --- |
| **Demographics** |  |
| Total number of patients | 11 |
| Male sex, n (%) | 11 (100) |
| Age (years), median (range) | 64 (52 – 74) |
| **Primary tumor** |  |
| Location (gastric cardia/EGJ), n (%) | 11 (100) |
| **Histology, n (%)** |  |
| Moderately differentiated adenocarcinoma | 1 (9) |
| Poorly differentiated adenocarcinoma | 5 (46) |
| Adenocarcinoma (NOS) | 5 (46) |
| **Intramural metastasis** |  |
| Multiple metastases, n (%) | 6 (55) |
| Distance from primary tumor (mm), range | 20–80 |
| **Treatment** |  |
| Radical esophagectomy + gastrectomy, n (%) | 8 (73) |
| Preoperative chemotherapy, n (%) | 2 (18) |
| **Prognosis** |  |
| Lymph node metastasis present, n (%) | 9 (82) |
| Overall mortality, n (%) | 9 (82) |
| Median overall survival (months), range | 8.5 (1.5 – 20) |

**Supplementary Table S4. Descriptive summary of clinical characteristics and outcomes of 11 patients with esophageal intramural metastasis from EGJ adenocarcinoma**

Data are presented as n (%), median (range), EGJ, esophagogastric junction.
