# Supplementary material for: Esophageal intramural metastasis from adenocarcinoma of esophagogastric junction: a case report and literature review
Source: Front Oncol. 2026 May 11;16:1792292. doi: 10.3389/fonc.2026.1792292 (PMC13199032; doi:10.3389/fonc.2026.1792292)
Supplement: Supplementary file 5 [file Table5.docx]

| **Author (Year)** | **Patient information** | **Clinical findings** | **Diagnostic assessment** | **Therapeutic interventions** | **Follow-up outcomes** | **Discussion quality** | **Informed consent** | **Patient perspective** | **Overall quality** |
| --- | --- | --- | --- | --- | --- | --- | --- | --- | --- |
| Gupta 2023^[15]^ | Comprehensive | Not described | Thorough | Well-documented | Reported | Relevant | Obtained | Not mentioned | Moderate |
| Kitano 2026 ^[13]^ | Comprehensive | Detailed | Thorough | Well-documented | Reported | Relevant | Obtained | Not mentioned | High |
| Wang 2025 (Case 1 & 2)^[14]^ | Comprehensive | Not described | Thorough | Well-documented | Reported | Relevant | Obtained | Not mentioned | Moderate |

**Supplementary Table S5 Quality assessment of included case reports on intramural metastasis in esophageal squamous carcinoma based on the CARE guidelines**
