# Supplementary material for: Esophageal intramural metastasis from adenocarcinoma of esophagogastric junction: a case report and literature review
Source: Front Oncol. 2026 May 11;16:1792292. doi: 10.3389/fonc.2026.1792292 (PMC13199032; doi:10.3389/fonc.2026.1792292)
Supplement: Supplementary file 6 [file Table6.docx]

| **Case** | **Reference** | **Age/Sex** | **Histology** | **No. of mets** | **Location** | **LN mets** | **Treatment** | **Survival (mo)** | **Outcome** |
| --- | --- | --- | --- | --- | --- | --- | --- | --- | --- |
| 1 | Gupta 2023 ^[15]^ | 59/F | SCC | 1 | Gastric cardia | Yes | Radical esophagectomy | — | — |
| 2 | Kitano 2026 ^[13]^ | 73/M | SCC | 1 | Lesser curvature (gastric angle) | Yes | Chemoimmunotherapy (CF + pembrolizumab) → conversion surgery | 8+ | Alive, recurrence-free |
| 3 | Wang 2025 (Case 1) ^[14]^ | 76/M | SCC | 1 | Cardia-fundus | Yes | Chemotherapy + immunotherapy (sintilimab + cisplatin + paclitaxel) | 7+ | Partial remission |
| 4 | Wang 2025 (Case 2) ^[14]^ | 66/M | SCC (II–III) | 1 | Gastric fundus | No | Partial esophagectomy + gastrectomy | — | Lost to follow-up |

**Supplementary Table S6. Clinical characteristics, metastatic patterns, treatment modalities, and outcomes of individual patients with intramural metastasis in esophageal squamous carcinoma**
