# Supplementary material for: Esophageal intramural metastasis from adenocarcinoma of esophagogastric junction: a case report and literature review
Source: Front Oncol. 2026 May 11;16:1792292. doi: 10.3389/fonc.2026.1792292 (PMC13199032; doi:10.3389/fonc.2026.1792292)
Supplement: Supplementary file 7 [file Table7.docx]

**Supplementary Table S7. Clinical characteristics of patients with intramural metastasis in the esophageal squamous cell carcinoma (ESCC) group**

| **Characteristic** | **Summary statistic** |
| --- | --- |
| **Demographics** |  |
| Total number of patients | 4 |
| Male sex, n (%) | 3 (75.0) |
| Female sex, n (%) | 1 (25.0) |
| Age (years), median (range) | 67.5 (59–76) |
| **Primary tumor** |  |
| Location (middle/lower thoracic esophagus), n (%) | 4 (100) |
| **Histology (SCC), n (%)** |  |
| Well-differentiated | 0 (0) |
| Moderately differentiated | 0 (0) |
| Poorly differentiated | 0 (0) |
| **SCC (NOS)** | 4 (100) |
| Intramural metastasis (gastric) |  |
| Solitary metastasis, n (%) | 4 (100) |
| Multiple metastases, n (%) | 0 (0) |
| **Location in stomach, n (%)** |  |
| Cardia | 2 (50.0) |
| Fundus | 1 (25.0) |
| Lesser curvature | 1 (25.0) |
| **Treatment** |  |
| Surgery alone (esophagectomy + gastrectomy), n (%) | 2 (50.0) |
| Chemotherapy + Immunotherapy alone, n (%) | 1 (25.0) |
| Chemoimmunotherapy, conversion surgery, n (%) | 1 (25.0) |
| Lost to follow-up, n (%) | 0 (0) |
| **Prognostic factors** |  |
| Lymph node metastasis positive, n (%) | 3/4 (75.0) |
| Lymphatic invasion positive, n (%) | 0/4 (0) |
| **Survival (available data, n=**4) |  |
| Overall mortality, n (%) | 0 (0) |
| Alive without recurrence, n (%) | 3 (75.0) |
| Alive with partial remission, n (%) | 1 (25.0) |
| Median follow-up (months), range | 7.5 (7–8) |
