# Supplementary figures and images for: Esophageal intramural metastasis from adenocarcinoma of esophagogastric junction: a case report and literature review
Source: Front Oncol. 2026 May 11;16:1792292. doi: 10.3389/fonc.2026.1792292 (PMC13199032; doi:10.3389/fonc.2026.1792292)

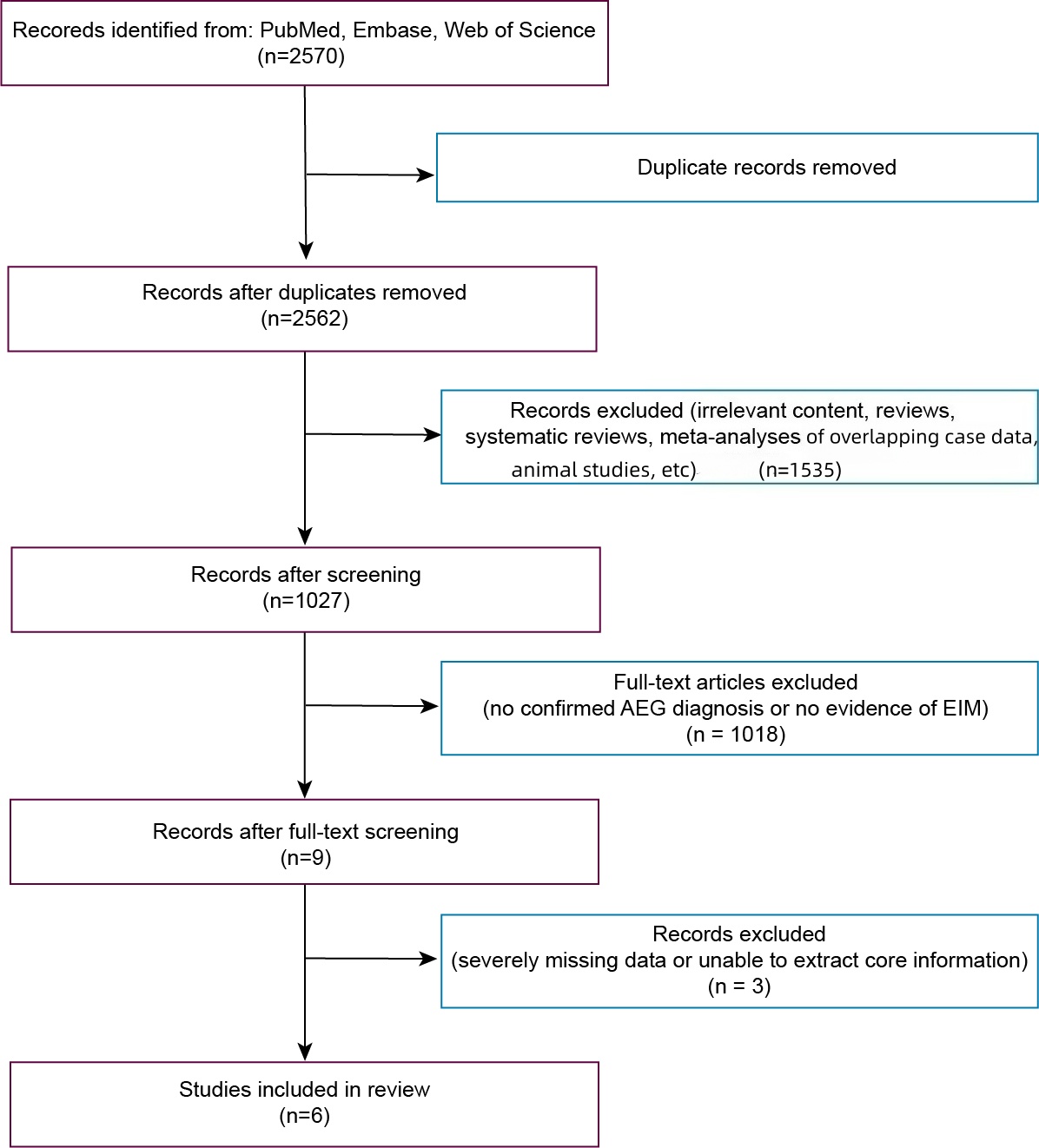

Supplement: Supplementary file 8 [file Image1.jpeg]
